# p16 Expression in Laryngeal Squamous Cell Carcinoma: A Surrogate or Independent Prognostic Marker?

**DOI:** 10.3390/pathogens13020100

**Published:** 2024-01-24

**Authors:** Roberto Gallus, Davide Rizzo, Giorgia Rossi, Luca Mureddu, Jacopo Galli, Alberto Artuso, Francesco Bussu

**Affiliations:** 1 Otolaryngology, Mater Olbia Hospital, 07026 Olbia, Italy; roberto.gallus@materolbia.com (R.G.); alberto.artuso@materolbia.com (A.A.); 2 U.O.C. Otorinolaringoiatria, Azienda Ospedaliero Universitaria di Sassari, Viale San Pietro, 43, 07100 Sassari, Italy; drizzo@uniss.it (D.R.); fbussu@uniss.it (F.B.); 3 Otolaryngology Division, Department of Medicine, Surgery and Pharmacology, University of Sassari, Viale San Pietro, 43, 07100 Sassari, Italy; 4 Unit of Otorhinolaryngology and Head-Neck Surgery, “A. Gemelli” Hospital Foundation IRCCS, 00168 Rome, Italy; giorgia.ros@tiscali.it (G.R.); jacopo.galli@unicatt.it (J.G.); 5 Department of Head-Neck and Sensory Organs, Catholic University of Sacred Heart, 00168 Rome, Italy

**Keywords:** p16^ink4a^ overexpression, p16^ink4a^ inactivation, LSCC, HPV, prognostic marker

## Abstract

Laryngeal squamous cell carcinoma (LSCC) is a common malignancy that, despite scientific advancements, has not seen an improvement in its prognosis in the last decades. Few promising predictive markers have been found and none are relevant in clinical practice. p16^ink4a^, an oncosuppressor protein involved in cell cycle arrest, with a prognostic impact on other cancers, has been widely used in the head and neck region as a surrogate marker of HPV infection. Published papers and recent meta-analyses seem to minimize the biological role of HPV in the context of LSCC’s cancerogenesis, and to disprove the reliability of p16^ink4a^ as a surrogate prognostic marker in this context, while still highlighting its potential role as an independent predictor of survival. Unfortunately, the available literature, in particular during the last two decades, is often not focused on its potential role as an independent biomarker and few relevant data are found in papers mainly focused on HPV. The available data suggest that future research should focus specifically on p16^ink4a^, taking into account both its potential inactivation and overexpression, different patterns of staining, and immunohistochemistry cutoffs, and should focus not on its potential role as a surrogate marker but on its independent role as a predictor of survival.

## 1. Introduction

The discovery and study of the role of high-risk Human Papillomaviruses (HPVs) in oropharyngeal squamous cell carcinomas (OPSCCs) during the last 20 years have completely changed our understanding of a subset of these tumors and have supported the identification of HPV-positive OPSCC as a distinct clinical entity [1,2,3,4,5,6]. The knowledge accumulated during this timeframe has led to the recent change in the American Joint Committee on Cancer (AJCC) TNM (tumor, node, metastasis) staging system and is supposed to ultimately pave the way to deintensification protocols in a selected cohort of patients [7]. An unintended byproduct of the rise in HPV-positive OPSCCs has been the shedding of light on the elected surrogate marker p16^INK4a^ [8], which is well known to oncologists and clinicians dealing with cervical cancers and less known to otolaryngologists and researchers in the field of head and neck cancers. In fact, in both the aforementioned eighth edition of the AJCC TNM staging system and current major guidelines, p16^INK4a^ immunohistochemistry (p16 IHC) is considered the reference technique for the detection of HPV in the oropharynx [9,10]. However, this technique has some relevant limitations that have been extensively discussed elsewhere [11,12].

After first confirming the role of HPV in the cancerogenesis of a subset of OPSCCs, efforts were made to seek its impact on other head and neck cancers, with mixed and sometimes disappointing results, especially when it came to its impact on prognosis [13]. Many of the studies that focus on non-oropharyngeal cancers have often relied, probably inappropriately [14], on p16^INK4a^ as a surrogate marker of HPV involvement, thereby gifting us with a relevant amount of data regarding its overexpression in squamous cellular carcinomas (SCCs) from different subsites of the head and neck. These same studies have sometimes offered data on its eventual independent prognostic significance in these subsites. The aims of this narrative review are to discuss such evidence, often feeble, in the setting of laryngeal squamous cell carcinomas, and to put the attention front and center on p16^INK4a^, which is often a misunderstood marker that is discussed prevalently in relation to HPV in this particular setting.

## 2. p16^ink4a^

p16 is a tumor suppressor gene located within the INK4a/ARF locus on the short arm of chromosome 9p21.3 that encodes for the homonym protein p16^INK4a^ and three other transcriptional variants: p14ARF (alternative reading frame), p12, and p16γ [15]. Other names of the same gene include MTS-1 (multiple tumor suppressor-1), INK4A (inhibitor of cyclin-dependent kinase 4a), and CDKN2A (cyclin-dependent kinase inhibitor 2a) [16,17,18]. p16^INK4a^ belongs to the INK4 family of CDK inhibitors that also includes p15^INK4B^, p18^INK4C^, and p19^INK4D^. All these proteins are involved in tumor growth inhibition and tumor suppression [19,20]. The p16 gene function in particular is related to the regulation of the p53 and pRB cell cycle pathways [15]. Its location (9p21.3) is the site of the loss of heterozygosity in many malignancies [19]. The gene includes five exons (E1β, E1α, E2, E2γ, and E3), and three of them (E1α, E2, and E3), each encoding for 156 amino acids, are responsible for the p16^INK4a^ transcript. The structure of p16^INK4a^ includes four ankyrin repeats arranged in a helix involved in interactions with target proteins [21]. 

The p16^INK4a^ involvement in cell cycle regulation is focused on the inhibition of the S phase. While its function is still not fully understood, what is known is that p16^INK4a^ is able to bind CDK4/6 and to consequently inhibit D–CDK4/6 complex formation and the phosphorylation of Rb family members. Hypophosphorylated Rb proteins bind E2F1, preventing the transcription of the S-phase genes and leading to cycle arrest [19]. p16^INK4a^ and pRb seem to be bound by a feedback loop in which hypophosphorylated pRb downregulates p16^INK4a^, while phosphorylated pRb induces p16 expression [22]. p16^INK4a^ overexpression is at least partially involved in cellular senescence and cell cycle arrest in nonsenescent cells [23], a growth arrest mechanism meant to protect the cell from hyperproliferative mechanisms and cellular stress (such as exposure to toxic substances, oxidative stress, DNA damage) [24]. It should be noted that other mechanisms can regulate p16^INK4a^ expression, and that its oncosuppressor role can be mediated via different mechanisms not limited to the pRb–E2F pathway [25,26,27,28]. p16^INK4a^ also seems to be involved in other cellular mechanisms potentially relevant for tumorigenesis, acting as a tumor suppressor on various fronts. In fact, in vivo and in vitro data suggest a role in tumor invasion, angiogenesis, and apoptosis in various cancers [29]. Other reported functions of p16^INK4a^ exerted through an accurate balance between its overexpression and downregulation include a potential role in the healing processes [30,31,32], stem cell maintenance and cell differentiation [33], glucose homeostasis and fat metabolism [34,35], and embryonic development [36].

Both the downregulation and overexpression of p16^INK4a^ are phenomena seen in the context of cancer [29]. Available data show that p16^INK4a^ is inactivated via various mechanisms (point mutations, loss of heterozygosity, methylation of the promoter region) in 50% of human cancers, including head and neck cancers [19]. Mutations at the level of the INK4a/ARF locus are seen in some inherited cancer syndromes [37,38,39]. Available evidence shows that the inactivation of p16^INK4a^ could be both an early event leading to preneoplastic lesions or a late event during tumor progression [40,41,42]. While the inactivation of p16^INK4a^ is usually seen in premalignant and malignant lesions and has been interpreted as a step to overcome the natural protection mechanism of the cell, overexpression too can be seen in benign lesions and in the very early stages of transformation, and sometimes also in malignant lesions. Overexpression in benign and premalignant lesions has been interpreted in the context of its natural role as an oncosuppressor and mediator of oncogene-induced senescence [19], protecting the cell from malignant transformation, while its accumulation in the later phases of malignant transformation could be related to anomalies of the p16Ink4a–Rb pathway and to accumulation through a positive-feedback mechanism [43]. This interpretation is consistent with the fact that Rb loss is an event frequently seen in tumor progression. Unfortunately, little is known about p16^INK4a^ overexpression in cancer, a phenomenon seen especially in HPV-related cancers, such as cervical cancer, anal cancer, or oropharyngeal squamous cell carcinoma, albeit sometimes described in non-HPV-related cancers [44,45,46,47]. Interestingly, while the main known function of p16^INK4a^ takes place in the nucleus, its overexpression is often seen at the cytoplasmic level, possibly with different functions. While the exact mechanisms behind cytoplasmic accumulation are poorly understood, both CDK4 sequestration and anion exchanger 1 (AE1)-mediated accumulation have been hypothesized [48,49]. A prevalent cytoplasmic or concomitant nuclear/cytoplasmic expression has been seen in the contexts of breast cancer, colorectal cancer, and some non-epithelial tumors [44,45,48,50,51,52,53]. Interestingly, a high cytoplasmic and low nuclear expression has been linked with a poorer prognosis in head and neck cancers [54].

### 2.1. p16^ink4a^ as a Surrogate Marker

In the last decades, immunostaining for p16^INK4a^ has seen a great deal of success as a diagnostic technique in cervical cancer and OPSCC, which are both HPV-related tumors [55]. In fact, in this particular setting, p16^INK4a^ behaves as a surrogate marker not only of HPV infection but also of its active role in the cancerogenic process [56]. The reason behind the overexpression of p16^INK4a^ in HPV-related cancers has been extensively discussed in the literature. In brief, after the integration of the high-risk HPV genome into the host cell, three main oncoproteins, E5, E6, and E7, are expressed. E7 is able to associate with pRb and the related proteins p107 and p130, leading to their degradation, ultimately unlocking cell cycle progression by allowing E2F-regulated transcription [57]. This event leads to p16^INK4a^ overexpression via a positive-feedback loop. Part of the reason that p16 immunohistochemistry (IHC) has been extensively used lies in the fact that it is a simple, broadly available, and relatively cheap technique [11], and despite some relevant limitations, as mentioned before, it is now considered the reference technique for the identification of HPV-related OPSCCs by the major guidelines, albeit with some caveats [58]. In fact, while it is acceptable to use p16 ICH in populations characterized by high fractions of HPV-positive OPSCCs, such as those of North America and Northern Europe, the same is not true elsewhere. While p16 IHC is characterized by a high sensibility, it lacks specificity, which means that in populations with a low prevalence of HPV-positive OPSCCs, higher numbers of false-positive cases will be seen. This has a potential detrimental effect on patient stratification and selection for deintensification protocols in these populations, and it could, theoretically, also affect HPV detection in subsites other than the oropharynx, where the HPV reported prevalence is often low. In fact, an ever-growing amount of evidence discourages its use as a surrogate marker of HPV infection outside of the oropharyngeal site and, in particular, in LSCCs, as well as under specific conditions (a low prevalence of HPV-related oropharyngeal squamous cell carcinoma), even for the oropharyngeal site [5,14,59,60,61,62]. The last decade of research on the topic seems to also minimize its potential biologic role in the cancerogenic process of LSCCs, probably impacting only a small subgroup of patients. This subset is quantifiable in around 11% of LSCCs according to a recent analysis of the National Cancer Database that notably included data coming from studies that sometimes used p16IHC as the only detection method, thereby probably overestimating the true prevalence [63]. Notably, a recent meta-analysis by Sahovaler and colleagues also found a prognostic impact only when evaluating p16^ink4a^ positivity but not HPV DNA positivity [13], reflecting the potential different biological meanings of the two markers.

### 2.2. Prognostic Role of p16^ink4a^

As seen, under different circumstances, both p16^INK4a^ overexpression and downregulation can be seen in different cancers in both the early and advanced stages. When p16^INK4a^ is downregulated as a step towards cancerogenesis, lower expression levels can be a marker of both malignant transformation and a poor prognosis. This is the case with melanoma, in which lower expression levels are associated with metastases [64] and osteosarcoma, where it predicts a poor response to chemotherapy [65]. In the setting of pulmonary squamous cell carcinoma, p16 methylation is not only an early-stage event and potential diagnostic biomarker but also serves to differentiate a primary pulmonary lesion from a metastasis from a p16^INK4a^ -overexpressing cancer, such as cervical squamous cell carcinoma [66]. p16^INK4a^ downregulation has also been identified as a potential prognostic biomarker of a poor prognosis in non-small-cell lung carcinoma [66,67] and glioblastoma [68]. In tumors that overexpress p16^INK4a^ after malignant transformation, detecting this event could be of prognostic value. In fact, some studies suggest that p16^INK4a^ overexpression could be related to unfavorable tumor characteristics in a subset of tumors, including adenocarcinoma, breast cancer, astrocytoma, and gastrointestinal stromal tumors [44,45,47,69].

### 2.3. p16^ink4a^ in the Context of LSCC

Laryngeal squamous cell carcinoma is a common head and neck cancer. It affects men more commonly than women. Its known risk factors are mainly tobacco and alcohol consumption, but dietary and environmental factors may also be involved, and the potential role of laryngopharyngeal reflux has been discussed [70]. LSCC is one of the rare cancers with a decreased 5-year survival rate during the last decades [63], and, for this reason, prognostic and predictive markers for LSCC are particularly sought-after. While no prognostic markers are routinely used or recommended in clinical practice for LSCC, a number of potential prognostic markers have been proposed over time, and a comprehensive review has recently been published by Cavaliere and colleagues [71]. The role of HPV infection in cancerogenesis and as a prognostic biomarker is much more controversial, and although a small subset of LSCC may be related to HPV, its clinical and prognostic relevance is probably limited [13,63]. However, most of the available data on p16^INK4a^ expression in LSCC derive from the use of p16IHC as a common technique of choice to demonstrate HPV involvement, often dismissing the possibility of overexpression related to different mechanisms.

By analyzing the literature, two distinct phases of the research effort covering the role of p16^ink4a^ in laryngeal cancer can be identified. The first one, between the 1990s and the turn of 2000, was more focused on the role of P16 inactivation as a step toward cancer progression, and the second phase, which started around the mid-2000s, was more focused on the role of p16^ink4a^ as a surrogate marker of HPV infection and on the role of its overexpression. In fact, almost 30 years ago, in the wake of works defining the inactivation of p16^ink4a^ as a potential step towards cancerogenesis in some head and neck cancers [72,73], the first evidence of the loss of heterozygosity (LOH) at the 9p arm in a subset of laryngeal cancers in a variable percentage of LSCCs emerged [74,75]. Later, along with allelic deletions, both point mutations and promoter hypermethylation were described as relatively frequent events involved in a subset of LSCCs, and LOH in particular seemed to be associated with more advanced and metastatic cases [76]. During this first wave of relevant papers, enough evidence was building around the role of the inactivation of p16^ink4a^ in head and neck cancers to suggest gene therapy as a potential treatment to be considered [77,78]. Notably, p16^ink4a^ inactivation seemed to be present in more than 50% of patients [79]. The role of the LOH and mutations in head and neck cancers was later confirmed via next-generation sequencing analysis [80,81]. It has been suggested that the length of exposure to tobacco and alcohol (but not the intensity) is associated with the homozygous deletion of p16 [82], and that a lack of dietary folates is associated with p16 methylation [83]. An association between weak p16^ink4a^ expression and advanced disease was later confirmed by other authors [79,84], while p16 point mutations were found to be independently associated with the risk of relapse and death in advanced LSCCs, albeit only in a small subset of them [85]. In contemporary papers, p16 anomalies were also found to be potentially associated with the tumor grade [86,87] and invasiveness and regional lymph node metastasis [56,88,89,90]. However, not all research groups looking for a prognostic impact of p16 inactivation found the same results, sometimes confirming a higher frequency of genetic anomalies in more advanced cases [79,91,92,93]. In later years, characterized by papers focused on the potential role of HPV in non-oropharyngeal head and neck cancers, some papers started to point to the fact that while a subset of laryngeal cancers indeed overexpressed p16^ink4a^ (range: 4.7% [94]–39.02% [95], according to the papers cited in this review), this did not seem to reflect relevant HPV involvement in the cancerogenic process, but it was still associated with a trend towards better survival [94,96,97,98] and progression-free survival [94,97]. Relevant exceptions exist at both extremes. In a series of 123 glottic LSCCs, p16-positive cases had a significantly better 2-year disease-free survival and fewer nodal relapses [95]. Allegra et al. found a positive impact on the 5-year overall survival (OS) and disease-specific survival in primarily operated cases, along with fewer nodal metastases [99]. A similar result was found by our group analyzing 95 consecutive LSCCs treated with different modalities. We found a positive impact on the relapse-free survival (RFS) for the whole series and a positive impact on the OS in primarily operated cases [100]. In a study involving 812 patients, Zhu and colleagues found that p16^ink4a^-positive patients had better OS, disease-specific survival, and RFS [101]. Other studies failed to find any trends or significant correlations between the p16^ink4a^ status and prognosis. Young and colleagues did not find any impact on the 2-year OS or RFS in a cohort of 307 patients, and other groups reported similar results for smaller cohorts [102,103]. Lastly, the paper by Larque and colleagues is a relevant outlier, as they found a better prognosis in patients with a negative p16^ink4a^ status. Notably, they also looked for p16^INK4a^ mRNA expression and gene mutations that did not correlate well with p16^INK4a^ protein expression [104]. Two recent papers, one a propensity-scored analysis of the National Cancer Database for survival outcomes by high-risk Human Papillomavirus status in non-oropharyngeal head and neck squamous cell carcinomas [63], and the other a systematic review and meta-analysis of the survival outcomes in Human Papillomavirus-associated non-oropharyngeal squamous cell carcinomas [13], offer us a broad view of the issue and some interesting insights. Tian and colleagues included in their analysis a total of 4804 LSCC patients, and an HPV+ status was associated with better survival at 1, 2, and 5 years of follow-up. A relevant issue with this paper is that it is focused on the HPV status, meaning that some papers, albeit a minority, did not use p16 to assess the HPV status and thus their analyses do not fairly reflect the impact of p16^INK4a^ on the prognosis. This limitation is overcome in the systematic review and meta-analysis by Sahovaler and colleagues, as they decided to group studies according to their detection techniques. Their paper included 24 studies and 9793 laryngeal cancer patients, and the subgroup analysis showed a significant survival improvement for p16^INK4a^-positive patients but not for HPV-DNA-positive patients.

What has been discussed up to this point has directly and indirectly highlighted some relevant problems that prevent us from fully understanding the impact of p16^INK4a^ on laryngeal cancer, and especially on its prognosis. As seen, few papers have been published on the matter, and they are often not focused on p16^INK4a^ but prevalently or solely on the role of HPV in laryngeal cancer [105,106]. Moreover, different “non oropharyngeal tumors” are often grouped together, determining a loss of precious data. The discussed papers analyze the impact of p16^INK4a^ on tumors of different stages and treated with different treatment modalities. This could be relevant, as all the papers discussing a significant impact on the prognosis were mainly based on patients treated with surgery as their primary option [95,99,100,101], and the papers that stratified according to the treatment modality found no impact [101] or a negative one [100] on patients treated with nonsurgical modalities. As seen, there are two strikingly distinct phases of the research efforts, one focused on p16^INK4a^ inactivation as a step of LSCC cancerogenesis with a potential negative impact on prognosis, and the other focused on p16^INK4a^ overexpression both as a marker of HPV involvement in LSCC cancerogenesis and as a potential positive prognostic marker in and of itself. The two conditions might very well coexist, and this fact should be reflected properly in future research efforts, as both possibilities should be sought while looking for the prognostic impact of p16^INK4a^ expression. One problem that is tightly linked to the last one is the cutoff chosen to determine p16^INK4a^ expression. The most relevant papers discussed in this review use a wide range of cutoffs, including the following: undefined [97,98]; expression (undefined level) in both the nucleus and cytoplasm [94]; >70% diffuse staining (nuclear and cytoplasmic) [95,101,107]; nuclear staining scored with the intensity reactivity score (IRS) with various cutoffs [99,100]; strong and diffuse (>25%) cytoplasmic and/or nuclear staining [102]; an intensity score of 2 (moderate) or 3 (strong) in ≥30% of tumor cells [103]; nuclear and cytoplasmic staining in >50% of cells [96]; nuclear staining in >50% of cells [108]; strong and diffuse cytoplasmic and nuclear staining in all basal and suprabasal cells [104]. This variability is detrimental to any attempt at a coherent analysis and interpretation of the data. It is not possible to tell by the state of the art how these different cutoffs affected the findings of the papers, or whether any of them correlate better with the clinicopathological characteristics or survival trends. Moreover, one thing to consider is the selection of a cutoff that reflects HPV positivity (a 70% cutoff with nuclear and cytoplasmic expression with at least moderate-to-strong intensity is recommended by the NCCN citing the guideline from the College of American Pathologists [109]), and another is a cutoff that reflects p16^INK4a^ overexpression or inactivation when considering it as an independent marker. The matter is further complicated by early evidence that the staining pattern of p16^INK4a^ might, in and of itself, be predictive of certain clinicopathological characteristics of the tumor [54,110]. Lazăr and colleagues [110] analyzed 88 cases of LSCCs looking for different patterns of the distribution/intensity of the staining and their respective correlations with the clinicopathological characteristics. They found that different patterns were associated with different levels of nodal involvement. A similar observation was made by Zhao and colleagues [54]. Future research focusing on p16^INK4a^ as a prognostic marker will need to properly assess the ideal cutoffs and analyze different staining patterns and their respective associations with the clinicopathological characteristics and survival outcomes.

## 3. Conclusions

The current body of research is limited and often out of focus. However, especially as clinicians that deal everyday with patients and challenging decisions, we are in desperate need of a prognostic marker, or, even better, a marker that is predictive of the treatment response, capable of mirroring in LSCCs the role of HPV in OPSCCs. The detection of p16^INK4a^ expression via IHC is a good candidate, as it is relatively cheap and accessible and can be applied on paraffin-embedded and formalin-fixed tissues, and it is only limited by its dependance on an experienced anatomopathologist for its interpretation. While its role as a surrogate marker of HPV infection has been largely re-evaluated in the context of LSCCs, and the role of HPV in the cancerogenesis of this particular subsite is probably marginal, it is our opinion that p16 may have some potential as a standalone prognostic marker. While, at the moment, this is only a hypothesis, we have seen that single studies, such as those published by Allegra and colleagues [99,110] and our research group [100], and a recent meta-analysis by Sahovaler and colleagues [13], point in this direction. This hypothesis could be better investigated in the setting of vast and multicentric prospective studies, accounting for both its under- or overexpression and different patterns of expression, to clarify once and for all its eventual prognostic significance and utility in clinical practice. Considering the potential relevance, the latter objective is worth a common scientific effort from the research groups most involved in the field.

## Data Availability

Not applicable.
